# *De novo* design and structure of a peptide-centric TCR mimic binding module

**DOI:** 10.1126/science.adv3813

**Published:** 2025-07-24

**Authors:** Karsten D. Householder, Xinyu Xiang, Kevin M. Jude, Arthur Deng, Matthias Obenaus, Yang Zhao, Steven C. Wilson, Xiaojing Chen, Nan Wang, K. Christopher Garcia

**Affiliations:** 1 Department of Molecular and Cellular Physiology, Stanford University School of Medicine; Stanford, CA 94305 USA; 2 Program in Immunology, Stanford University School of Medicine; Stanford, CA 94305 USA; 3 Department of Structural Biology, Stanford University School of Medicine; Stanford, CA 94305; 4 Howard Hughes Medical Institute, Stanford University School of Medicine; Stanford, CA 94305; 5 Department of Computer Science, Stanford University; Stanford, CA 94305

## Abstract

T cell receptor (TCR) mimics offer a promising platform for tumor-specific targeting of peptide-MHC in cancer immunotherapy. Here, we designed a *de novo* α-helical TCR mimic (TCRm) specific for the NY-ESO-1 peptide presented by HLA-A*02, achieving high on-target specificity with nanomolar affinity (K_d_ = 9.5 nM). The structure of the TCRm/pMHC complex at 2.05 Å resolution revealed a rigid TCR-like docking mode with an unusual degree of focus on the up-facing NY-ESO-1 side chains, suggesting the potential for reduced off-target reactivity. Indeed, a structure-informed *in silico* screen of 14,363 HLA-A*02 peptides correctly predicted two off-target peptides, yet our TCRm maintained peptide selectivity and cytotoxicity as a T cell engager. These results represent a path for precision targeting of tumor antigens with peptide-focused α-helical TCR mimics.

## Main text

Peptide-major histocompatibility complex (pMHC) molecules on cancer cells are important targets for antigen-specific solid tumor immunotherapy (*1, 2*). Class I MHC molecules present short peptide fragments (8–13 amino acids) from the intracellular proteome of a cell on its surface, providing a snapshot of the cell’s internal state and whether it is healthy, cancerous, or infected (*3, 4*). In humans, these molecules are encoded by the highly polymorphic human leukocyte antigen (HLA) genes, which determine which peptides are displayed to T cells. Cancer cells present mutant or self-peptides that are recognized by T cell receptors (TCRs) through a mechanism known as ‘MHC restriction’, which interact with both the MHC and the peptide composite surface (5). This recognition enables T cells to selectively detect and eliminate cancerous cells.

Therapeutic application of TCRs derived from the human immune system is hindered by their low affinity (*6, 7*). To overcome these limitations, many groups have engineered TCR mimic (TCRm) antibodies – high affinity molecules that replicate the MHC-restricted peptide recognition of TCRs (*8–14*). These mimics are valuable as cancer-specific probes and therapeutics. A recent example is tebentafusp-tebn, a bispecific T cell engager (TCE) approved in 2022 for uveal melanoma (*15, 16*). Despite these advantages, the discovery of TCR mimics remains challenging. Unlike TCRs, antibodies have not been naturally selected for the optimal balance of peptide versus MHC binding selectivity. As a result, engineered TCRm antibodies can interact more prominently with the MHC helices than the peptide, leading to off-target toxicity (*17–21*). Although strategies such as “repurposing” TCRm antibodies have been described (*8, 22*), fine-tuning these antibodies to have sufficient peptide specificity to use in therapies or probes is laborious and is limiting the impact of this approach (*17, 23–25*).

To address these obstacles, we sought to develop α-helical TCR mimics that could be rapidly programmed into cancer-specific probes or therapeutics. α-helical bundles offer several advantages over antibodies: they are compact, modular, and present amino acid side chains on a rigid platform (26), reducing the design complexity observed with flexible complementarity-determining region (CDR) loops on antibodies. Since CDR loop flexibility contributes to TCR cross-reactivity (*27–29*), a more rigid scaffold could help minimize structural adaptation to alternative peptides. Additionally, state-of-the-art protein design tools like RFdiffusion and ProteinMPNN (*30, 31*) favor α-helical folds and have demonstrated increased success rates for *de novo* protein design. We aimed to generate four-helix bundles, which are thermostable and known to mediate diverse protein-protein interactions across the natural proteome. Helical bundles, such as cytokines, are also ideal engineering scaffolds with proven robustness to amino acid substitutions along helical faces (33). By restricting our design space to this fold, we aimed to improve TCR mimic discovery rates using RFdiffusion and ProteinMPNN.

## Design of α-helical “mini”-TCR mimics with RFdiffusion and ProteinMPNN

As a first step, we generated 50 monomer folds (80–100 amino acids) with RFdiffusion and, by visual inspection, selected the most tightly packed helical bundles with a high likelihood of expression as recombinant proteins. We selected the fold deemed best matched to this criterion (99 amino acids) to be used as a template scaffold for RFdiffusion fold-conditioning over the peptide-MHC target. We selected the NY-ESO-1_157–165 (C9V)_ peptide presented on the HLA-A*02 allele as our model antigen, as it is a well-characterized tumor antigen (*2, 23, 32*) with structures in the Protein Data Bank (PDB) and robust experimental systems for downstream validation.

**Fig. 1.**
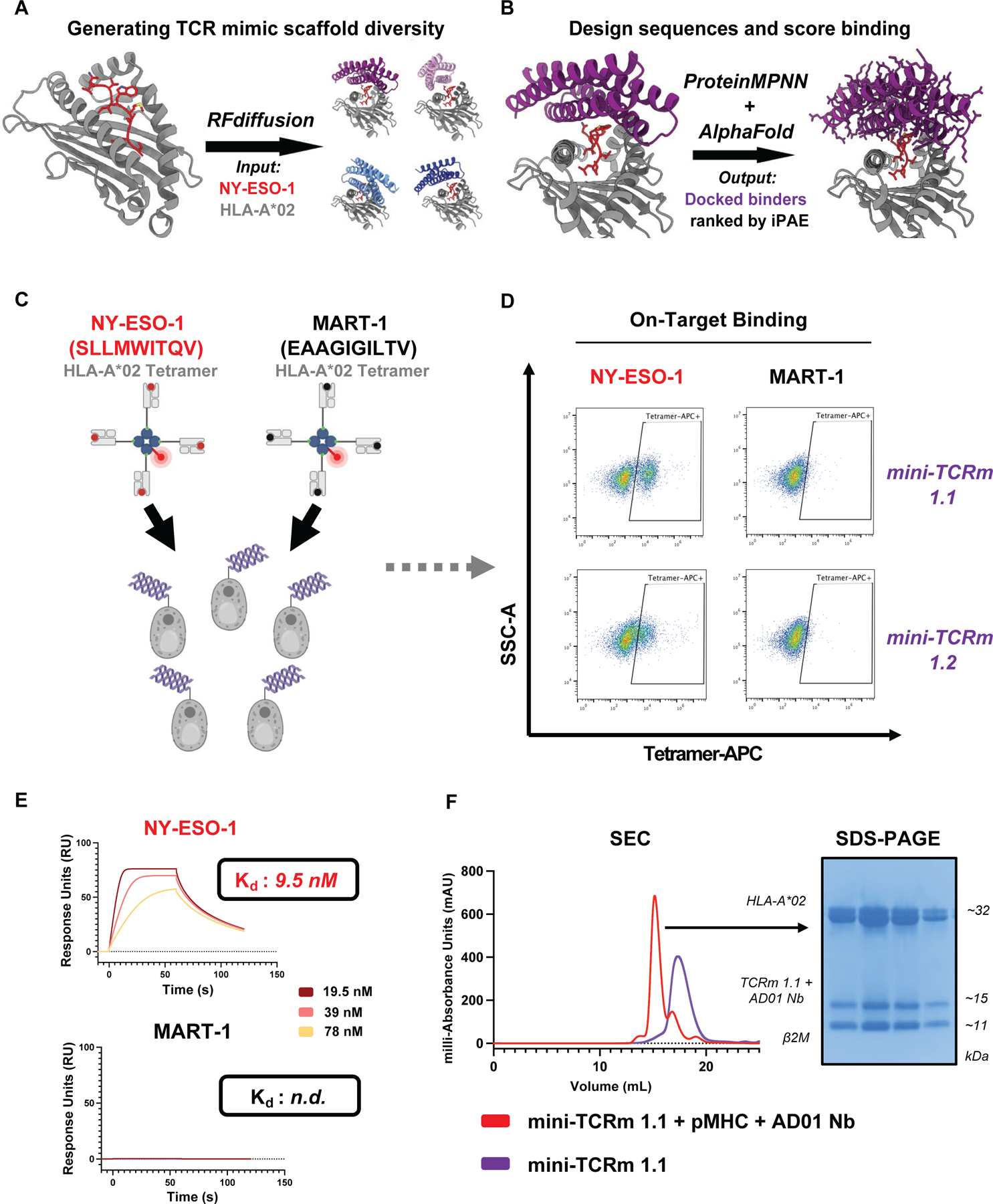
Design and experimental validation of *de novo* mini-TCR mimics. **(A)** Schematic of RFdiffusion fold-conditioning pipeline generating four hit scaffolds with the NY-ESO-1 HLA-A*02 input (red and gray) out of 100 distinct folds sampled. **(B)** Schematic of pipeline using ProteinMPNN to design 500 sequences for Scaffold #1 (purple) and predict likely binders with AlphaFold2. **(C)** Yeast display screening of top *de novo* designs with NY-ESO-1 (red) vs. MART-1 HLA-A*02 (black) tetramers. **(D)** Two of five evaluated binders staining NY-ESO-1 but not MART-1 HLA-A*02 tetramer by flow cytometry (see fig. S4A for gating strategy) (two independent experiments). **(E)** SPR analysis of mini-TCRm 1.1 analyte with immobilized NY-ESO-1 HLA-A*02 (red) vs. MART-1 HLA-A*02 (black). Dissociation constants indicated on sensograms (K_d_); n.d., not determined. **(F)** SEC profiles of mini-TCRm 1.1 (purple) vs. co-eluting complex (red) of mini-TCRm 1.1 with NY-ESO-1 HLA-A*02 and AD01 nanobody. SDS-PAGE gel showing four fractions of co-eluting complex pooled for crystal screens. kDa, kilodaltons.

Since crystal structures are not always available for a given pMHC antigen, we used AlphaFold (34) to predict the NY-ESO-1/HLA-A*02/β-2-microglobulin (β2M) structure, which together stabilize class I pMHC presentation. We specified the up-facing Met4, Trp5, Thr7, and Gln8 peptide residues as hotspots for design and masked scaffold loops to allow RFdiffusion to variably extend these regions during generation. We produced 100 distinct four-helix bundle folds docked over NY-ESO-1 HLA-A*02, each sampled for six amino acid sequences with ProteinMPNN, yielding 600 designs (data S1). AlphaFold2 then predicted each design docked with the target, and we ranked by their interaction predicted aligned error (iPAE) metric, as described in prior studies showing a correlation between iPAE values less than 10.0 and increased experimental success (35). iPAE measures AlphaFold’s confidence in the relative positioning of amino acids across a protein-protein interface, with lower values indicating greater design confidence. Of the 100 scaffolds, four yielded at least one design under an iPAE threshold of 10.0 and therefore, was considered a “hit” scaffold (Fig. 1A and data S1).

Our top scaffolds varied in helical bundle arrangement and size (99–129 amino acids), as well as peptide versus MHC contact profiles. To expand sequence variant diversity, we generated 500 sequences per hit with ProteinMPNN (2000 designs total) and scored each complex with AlphaFold2 (Fig. 1B and data S1). Scaffold #1 and #2 yielded the highest rates of passing the iPAE threshold (132/500 and 318/500, respectively), and upon visual inspection, we noticed desirable properties in Scaffold #1 over the other scaffolds. Notably, it adopted a centered, TCR-like docking mode over the peptide-MHC target, favoring peptide-specific contacts. Moreover, all four peptide hotspots were predicted contacts in several of the top designs. Scaffold #1 appeared to best meet our design criteria, and we proceeded to screen the lowest iPAE-scoring binders.

## Experimental validation of *de novo* TCR mimics specific to NY-ESO-1 HLA-A*02

The top five designs were displayed as single clones on yeast cells and stained with 100 nM NY-ESO-1 or MART-1 HLA-A*02 tetramer, with MART-1 serving as a negative control (Fig. 1C). We found two out of five designs stained the NY-ESO-1 tetramer but not MART-1 (Fig. 1D). We selected the strongest binder based on the highest mean fluorescence intensity (MFI) staining for NY-ESO-1 tetramer and designated it mini-TCRm 1.1. This binder was expressed as a recombinant protein in BL21 *E. Coli* and purified by Nickel-NTA and size-exclusion chromatography (SEC). The recombinant protein was soluble with high yields and eluted as a single SEC peak (Fig. 1F, purple). Surface plasmon resonance (SPR) analysis revealed a dissociation constant (K_d_) of 9.5 nM for NY-ESO-1 HLA-A*02, but no detectable affinity for MART-1 HLA-A*02 (Fig. 1E). This indicated that mini-TCRm 1.1 was peptide-specific for NY-ESO-1, with undetectable trace affinity for the same MHC presenting a different peptide.

For structural studies, we complexed mini-TCRm 1.1 with refolded NY-ESO-1 HLA-A*02 and the AD01 anti-β2M nanobody, which served as a crystallization chaperone. The complex co-eluted by SEC (Fig. 1F, red) and readily crystallized during screening.

## High-resolution crystal structure reveals peptide-specific interactions

**Fig. 2.**
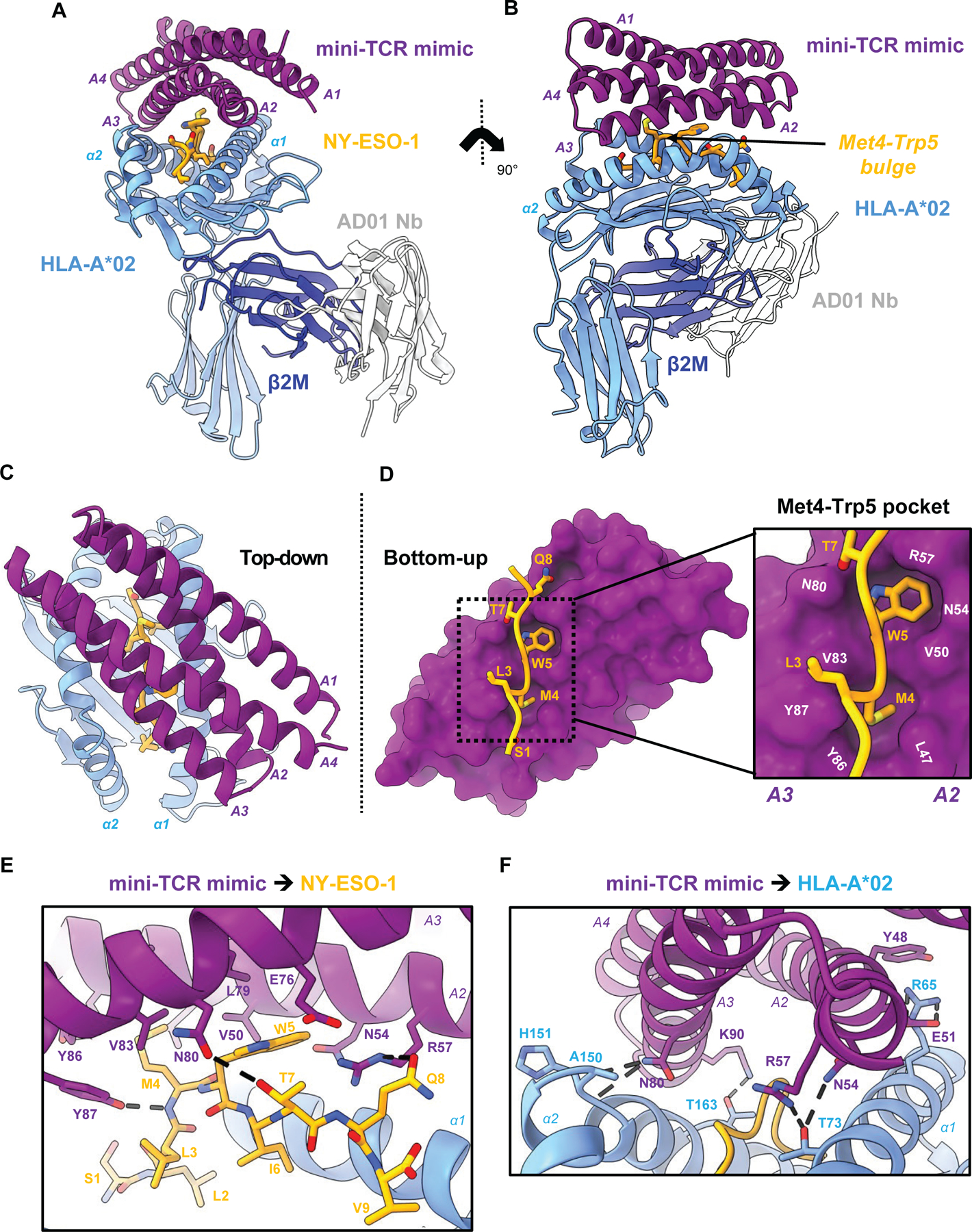
High-resolution crystal structure reveals peptide-specific interactions. **(A)** Front view of mini-TCRm/pMHC complex (PDB ID 9MIN). A1-A4 alpha-helices of mini-TCRm (purple); α1 and α2 helices of HLA-A*02 and full chain (light blue); NY-ESO-1 peptide (gold); β2M (dark blue); AD01 nanobody (white). **(B)** Side view of complex with Met4-Trp5 bulge on the NY-ESO-1 peptide. **(C)** Top-down view of complex. **(D)** Bottom-up view of peptide-specific interactions with surface of mini-TCRm (purple). Box zooming in on NY-ESO-1’s Met4-Trp5 motif (gold) fitting into mini-TCRm pockets formed between the A2 and A3 helices (key residue labels in white). **(E)** Hydrogen bonds (dashed lines) and key peptide-centric residues between mini-TCRm and NY-ESO-1 peptide. **(F)** Hydrogen bonds and salt bridges (dashed lines) between mini-TCRm and HLA-A*02.

We determined the crystal structure of the complex at 2.05 Å resolution (Fig. 2, A and B; table S1). The binder adopted a diagonal TCR-like docking footprint at a 40-degree angle relative to the peptide-MHC groove (Fig. 2C), burying 1216 Å^2^ of surface area with 31% from peptide-specific contacts (table S2). Buried surface area (BSA) refers to the portion of the protein interface that becomes inaccessible to solvent upon binding, indicating the extent of molecular interaction between two proteins. The mini-TCR mimic used 14 residues within the groove of the A2 and A3 helices to form a concentrated network of hydrophobic and polar peptide contacts. The peptide-contacting side chains bent inward creating a shape-complementary shell around the peptide side chains. In particular, the Met4-Trp5 peptide motif bulged prominently above the MHC groove and inserted into two hydrophobic pockets between the A2 and A3 helices (Fig. 2, B and D), stabilized by three hydrogen bonds (Fig. 2E). The binder also distributed hydrophobic contacts across both helices of HLA-A*02. These were supplemented by five hydrogen bonds and one salt bridge to the MHC, helping orient the binder precisely over the peptide (Fig. 2F). Three of these hydrogen bonds (formed by Asn54, Asn80, and Arg57 of the binder) interact with residues inside the HLA-A*02 groove around the peptide’s Trp5 residue.

Comparison of the crystal structure and AlphaFold model revealed general agreement in docking orientation and hydrophobic pocket formation around the Met4-Trp5 bulge (R.M.S.D 0.41 Å for 128 Cα atoms) (fig. S1A), albeit with substantial deviations in detail (table S3). Three differences affected peptide-specific contacts. AlphaFold predicted a hydrogen bond between Glu76 of the binder and Gln8 of NY-ESO-1, which was absent in the crystal structure (fig. S1B). Second, AlphaFold failed to predict a hydrogen bond between the binder’s Asn80 and the peptide’s Thr7, yet it was observed in the crystal structure (fig. S1C). Third, Arg57 of the binder made a hydrogen bond with the peptide’s Gln8 in the crystal structure, whereas AlphaFold predicted it to pair with Ile6’s backbone (fig. S1D). These pairwise bonding discrepancies in the predicted and actual structures underscore the value of structural validation in precisely refining *de novo* TCR mimic specificity.

## Comparison to existing antibody TCR mimic and TCR

**Fig. 3.**
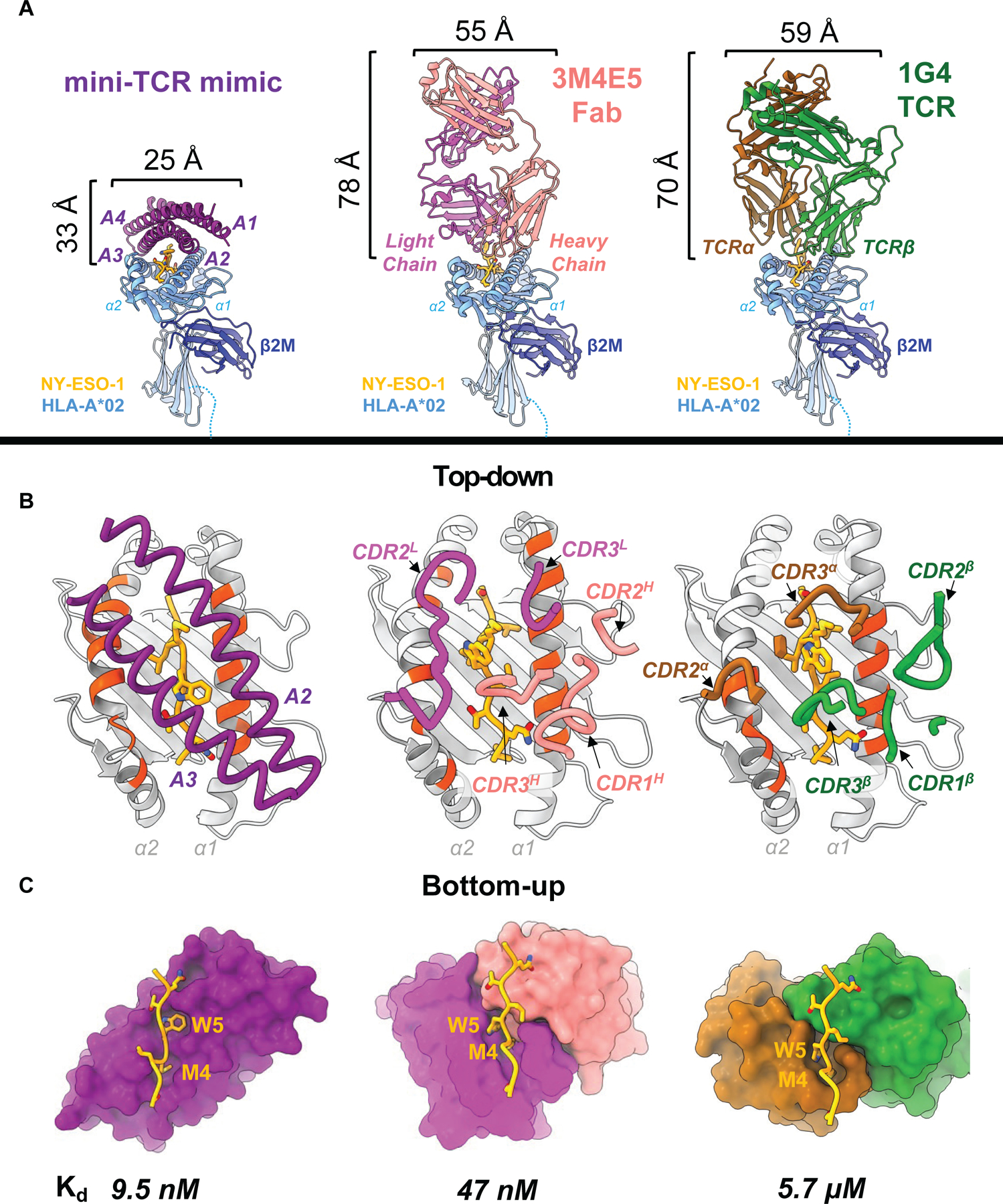
Comparison to existing antibody TCR mimic and TCR. **(A)** Front view comparison between mini-TCRm (purple), 3M4E5 Fab (magenta and salmon), and 1G4 TCR (brown and green) bound to NY-ESO-1 peptide (gold) and HLA-A*02 (light blue). Height and width of binders shown in Angstroms (Å). **(B)** Top-down comparison of docking footprints of each binder with MHC contact residues (red orange) and NY-ESO-1 peptide side chains (gold). **(C)** Bottom-up comparison of interactions between the NY-ESO-1 peptide (gold) and binding pockets, with the Met4-Trp5 motif labeled. Dissociation constants (K_d_) for each complex is shown at the bottom. nanomolar, nM; micromolar, μM.

We also compared the structure of the mini-TCR mimic to an NY-ESO-1-specific Fab (3M4E5 Fab – PDB: 3HAE) and TCR (1G4 TCR – PDB: 2BNQ) (*23, 32*). The mini-TCR mimic was compact, rigid, and less than half the height of the Fab or TCR (Fig. 3A). Unlike the Fab and TCR, which extended flexible loops into the MHC groove, our binder engaged the peptide’s upward-facing side chains through a groove between its helices (Fig. 3B). While the Fab and TCR used their CDR loops to create a large pocket simultaneously accommodating Met4 and Trp5 of the peptide, the designed binder used two shape-complementary pockets to capture them independently (Fig. 3C). Additionally, we compared binding footprints, identifying key contacts along the pMHC interface (Fig. 3B). The designed binder interacted with 19 MHC residues, compared to 14 for the Fab and 13 for the TCR. Nine residues on the MHC—Arg65, Lys66, Ala69, Gln72, Thr73, Val76 (α1 helix), and Lys146, His151, Gln155 (α2 helix)—were shared across all three footprints and are classical MHC “anchor residues” used by the germline-encoded CDR loops of TCRs (17). Our mini-TCR mimic buried the largest total surface area but the smallest proportion of peptide surface, relative to the Fab and TCR (table S2). Despite this, our mini-TCR mimic maintained peptide specificity and the highest binding affinity (Fig. 3C), suggesting that it formed focused peptide contacts while dispersing MHC interactions to stabilize binding. Furthermore, its rigid α-helical structure likely reduced the entropic penalty of binding by limiting conformational flexibility. Entropic penalty refers to the loss of freedom that occurs when a protein binds another protein, with smaller conformational rearrangements being more energetically favorable. In contrast, the Fab and TCR likely experienced greater entropic penalties with their flexible loops, which might contribute to less predictable cross-reactivity profiles.

## Structure-guided identification of off-target peptides from the human proteome

Using our crystal structure as a template, we evaluated the specificity of our mini-TCR mimic, considering two potential applications. First, as a diagnostic tool used to probe cell surface antigen expression on tumors for patient stratification, where broad off-target assessment and rapid peptide evaluation would suffice. Second, for therapeutic development, where understanding cross-reactivity informs the safety profile for clinical translation.

**Fig. 4.**
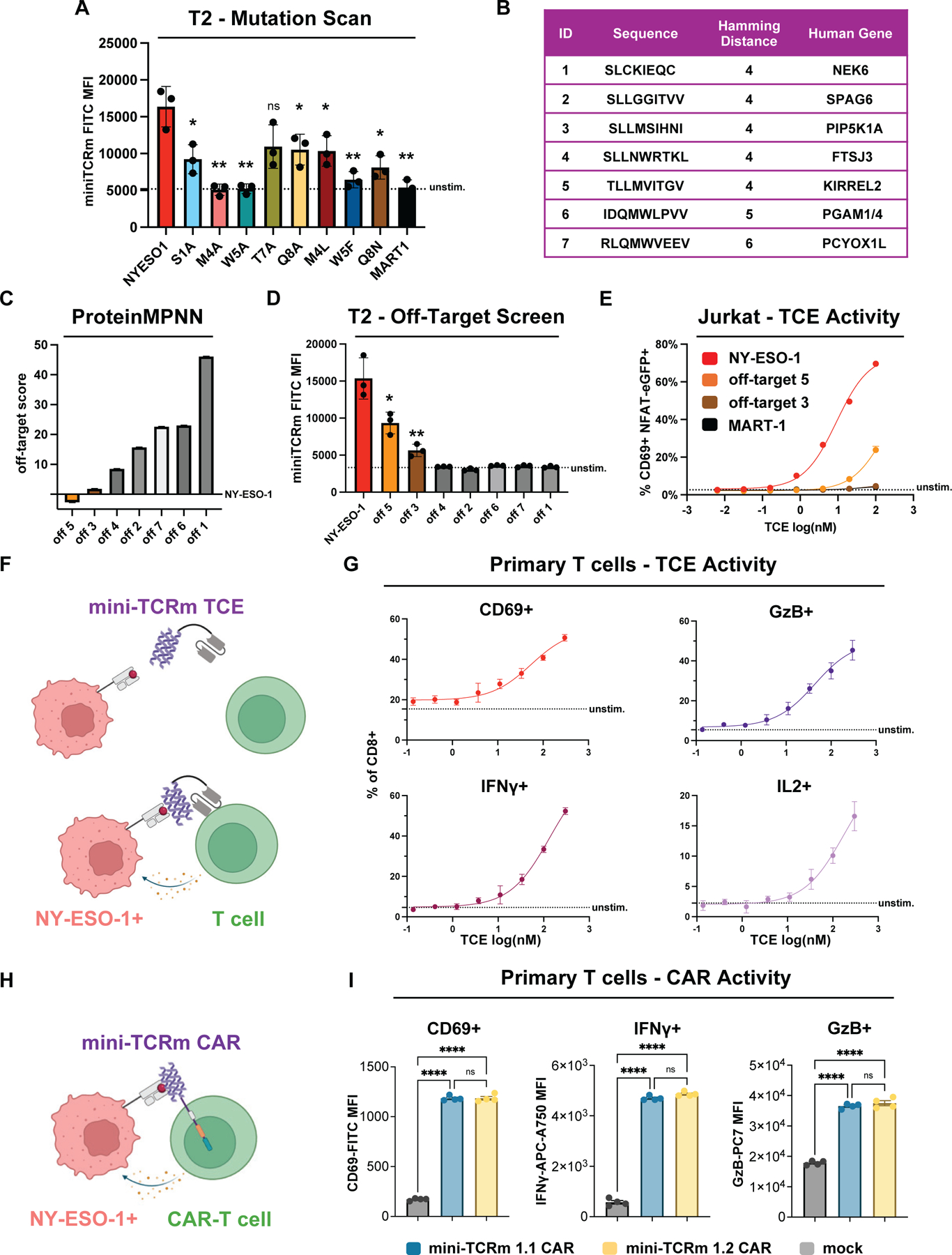
Mini-TCR mimic therapeutics exhibit peptide selectivity and cytotoxicity for NY-ESO-1 HLA-A*02. **(A)** Flow cytometry analysis of mini-TCRm staining T2 cells pulsed with NY-ESO-1 peptide variants (see data S2 for sequences; see fig. S4B for gating strategy). MART-1 and no peptide were used as negative controls. Data points represent the geometric MFI averaged across technical replicates per experiment (bars are mean ± SD, *n*=3, *N*=3). Statistical significance calculated relative to NY-ESO-1 (*P < 0.05, **P < 0.01, ns = not significant, unpaired Student’s t-tests). **(B)** Table of candidate off-targets identified from the MHC Motif Atlas. **(C)** Ranking of candidate off-targets based on ProteinMPNN and the crystal structure (see data S2 for scores). **(D)** Flow cytometry of mini-TCRm staining T2 cells pulsed with off-target candidates (see fig. S4B for gating strategy). Data points show the geometric MFI from technical replicates (mean ± SD, *n*=3, *N*=2). Statistical significance calculated relative to NY-ESO-1 (*P < 0.05, **P < 0.01, unpaired Student’s t-tests). **(E)** Jurkat CD69+ NFAT-eGFP+ reporter activation following co-culture with T2 pulsed with peptides and treated with mini-TCRm T cell engagers (see fig. S4C for gating strategy). Dose shown in log(nM) (dots are mean ± SD, *n*=3, *N*=2). **(F)** Schematic of a soluble mini-TCRm T cell engager (TCE; purple) activating T cells against an NY-ESO-1+ cell (salmon). **(G)** Cytotoxicity dose-response of mini-TCRm TCE in primary T cell co-cultures with NY-ESO-1-pulsed T2 cells (see fig. S4D for gating strategy). Data represent the percentage of cells positive for the cytotoxicity marker relative to negative controls (dots are mean ± SD, *n*=3, an independent experiment with lower peptide dosage yielded similar results though not counted as *N*=2). **(H)** Schematic of a mini-TCRm CAR-T cell (CAR; purple) targeting an NY-ESO-1+ cell (salmon). **(I)** Cytotoxic marker expression in mini-TCRm CAR-T cells co-cultured with NY-ESO-1+ A375 cells for 48 hours (see fig. S4E for gating strategy). Mock T cells were activated, untransduced controls. Data represent geometric MFI in CAR+ population (bars are mean ± SD, *n*=4, *N*=2). Statistical significance calculated for all pairs (****P < 0.0001, ns = not significant, one-way ANOVA followed by Tukey’s post-hoc test).

To confirm key peptide hotspots observed in the structure, we pulsed T2 cells with alanine-scanned NY-ESO-1 variants and stained with soluble mini-TCR mimic. Given its hydrophobic pockets, we hypothesized that other hydrophobic residues at peptide positions 4 and 5 could bind, so we included Leu4 and Phe5 mutants. Mutating Met4 or Trp5 to alanine abrogated binding, but Leu4 or Phe5 did not (Fig. 4A), confirming that the Met4-Trp5 bulge stabilized the complex, similar to the 3M4E5 single-chain variable fragment (scFv) (fig. S2). Thr7 and Gln8 alanine mutations had minimal effect when the Met4-Trp5 motif was present (Fig. 4A). Based on these findings, we anticipated that off-target peptides with the highest chemical similarity at NY-ESO-1_157–165 (C9V)_ peptide positions 1, 4, and 5 would be most likely to bind.

To test this, we conducted a Hamming distance search comparing the number of amino acid changes between the NY-ESO-1 peptide and 14,363 distinct HLA-A*02-presented 9-mer peptides detected by mass spectrometry from the MHC Motif Atlas (36). We restricted our search to this dataset, reasoning that MS detection provided a threshold for peptides found on the cell surface at sufficient quantities. After ranking the dataset (data S2), only five out of 14,363 peptides had the lowest Hamming distance of four mutations from NY-ESO-1 and were of human origin (Fig. 4B). All other peptides with five or greater Hamming distance were considered less likely to adopt NY-ESO-1’s target conformation and chemistry. A second Hamming distance search, which focused only on the critical Met4-Trp5 motif, found two additional peptides with an exact motif match but overall Hamming distances of five and six. Together, these seven peptides were selected for off-target analysis (Fig. 4B).

We explored whether ProteinMPNN could rank these peptides by their compatibility with our crystal structure. Using the NY-ESO-1 backbone as input and conditioning on the molecular context of the mini-TCR mimic and HLA-A*02, ProteinMPNN assigned an “off-target score” to each peptide, derived from its likelihood to fit with structural constraints. This score was calculated by subtracting each candidate off-target from NY-ESO-1’s likelihood. We hypothesized that more negative off-target scores (higher likelihoods to fit the structure) would identify real off-target peptides. Indeed, off-targets 3 and 5 exhibited the lowest scores (Fig. 4C), closely resembling NY-ESO-1, and were therefore predicted to bind the mini-TCR mimic. To confirm these predictions, we pulsed all peptides onto T2 cells and stained with our mini-TCR mimic. Consistent with the *in silico* analysis, cells pulsed with off-targets 3 and 5 bound the mini-TCR mimic, though more weakly than NY-ESO-1 (Fig. 4D).

Off-target 3 originated from PIP5K1A, a ubiquitously expressed kinase, while off-target 5 derived from KIRREL2, a transmembrane cell adhesion molecule predominantly found in pancreatic beta cells. AlphaFold-predicted structures of these peptides bound to HLA-A*02 revealed that the Met4 peptide residue contact was retained, along with a hydrogen bond between the mini-TCRm’s Tyr87 and the Met4 backbone, as well as a small polar residue at peptide position 1 (fig. S3). Interactions at peptide positions 5–8 varied slightly, subtly altering total BSA (table S2), which aligned with relative staining MFI in our T2 assay.

## Mini-TCR mimic therapeutics exhibit peptide selectivity and cytotoxicity for NY-ESO-1 HLA-A*02

We then determined whether our binder could function as a soluble peptide-specific T cell engager (TCE) by expressing and purifying it from mammalian cells. We designed our bispecific TCE as a fusion between the anti-CD3ε scFv from blinatumomab (37) and our mini-TCRm, connected by a short 5 amino acid Gly-Ser linker. Blinatumomab, the first FDA-approved bispecific TCE, activates T cells by forming a synthetic immune synapse upon binding both CD3ε and its target antigen. In our construct, the anti-CD3ε scFv recruits T cells to engage target cells displaying the peptide-MHC antigen (Fig. 4F).

We pulsed T2 cells with NY-ESO-1, MART-1, off-target 3, or off-target 5 and co-cultured in a 1:1 ratio with a Jurkat reporter cell line that expresses eGFP upon TCR signaling. A dilution series of our TCE was added and T cell activation was quantified the next day by measuring the percentage of CD69+ eGFP+ Jurkats via flow cytometry. In the presence of NY-ESO-1 but not MART-1 peptide, we observed effective T cell activation with an EC_50_ of 9.1 nM and nearly 70% of Jurkats activated with 100 nM engager (Fig. 4E). No activation was observed for off-target 3, while off-target 5 exhibited ~25% Jurkat activation with 100 nM engager but with a log-shifted EC_50_ of 174 nM. Between 1–10 nM, TCE activity was retained against the NY-ESO-1 peptide but not the off-target peptides, establishing a selectivity threshold. Our TCE also induced cytotoxic function in a dose-dependent manner when added to a co-culture of primary human T cells with NY-ESO-1-pulsed T2 cells (Fig. 4G). Additionally, both mini-TCRm hits 1.1 and 1.2 functioned in CAR-T cell format (Fig. 4, H and I), driving activation and cytotoxicity against the A375 human melanoma cell line, which endogenously expresses NY-ESO-1 and presents the NY-ESO-1_157–165_ peptide on HLA-A*02.

## Discussion

Here, we have shown that an alternative structural scaffold to antibodies offers a versatile approach for engineering peptide-specific TCR mimic probes and immunotherapies. By leveraging deep learning tools (*30, 31*), we accelerated the discovery of functional TCR mimics, generating 2,600 designs in 30 hours on a single graphics processing unit (GPU) and identifying two candidate binders in 1–2 weeks. These mini-TCR mimics have potential as cancer-specific diagnostics; for instance, when a new tumor antigen is identified, they could rapidly confirm its presentation on cancer cells, expediting antigen discovery.

For therapeutic applications, however, further development is required to eliminate off-target interactions. While *de novo* binders offer a more advanced starting point than traditional antibody screening, comprehensive off-target profiling remains essential before translation. A scalable approach could combine computational off-target predictions with high-throughput screening, such as yeast display of peptide-MHC complexes, to systematically prioritize and validate all potential cross-reactive peptides. If predictive approaches prove reliable, future mini-TCR mimics could be pre-optimized for specificity during the design process. This could make them particularly valuable for personalized cancer therapies, where tumor antigens from exome sequencing (*1, 2*) could guide real-time engineering of therapeutics in weeks to months.

Immunogenicity, or the ability to elicit anti-drug antibodies (ADAs), is another key consideration for *de novo* proteins. While ADAs can impact efficacy, many biologics remain effective due to dose adjustments or low neutralizing incidence. As synthetic proteins, mini-TCR mimics require empirical testing to assess the extent of immunogenicity. A proactive approach would involve computational epitope prediction and minimization of immunogenic T and B cell motifs, or engineering non-immunogenic human proteins.

Beyond specificity and immunogenicity, the α-helical platform offers engineering and design advantages over traditional antibodies. Mini-TCR mimics could be engineered into multi-specific or multi-valent biologics with greater structural precision in geometry or orientation than the conventional VH/VL heterodimer of antibodies. This capability could enable therapeutic formats that fine-tune antigen sensitivity or T cell function. Additionally, their structural rigidity enhances computational design accuracy, as the energetic consequences of mutations are more predictable than in flexible antibody loops (*26–29*). While crystallographic validation was crucial in this study, we anticipate that continued advancements in computational modeling will progressively replace the need for structural validation, such that it may not be necessary in future discovery efforts.

## Data and materials availability:

The crystallographic model and integrated intensities have been deposited in the RCSB PDB with accession code 9MIN. Raw diffraction images have been deposited in the SBGrid Data Bank (38). All other data needed to evaluate the conclusions are available in the manuscript and supplementary materials.

## Supplementary Material

Supplement

Data S1

Data S2

Materials and Methods

Figs. S1 to S6

Table S1 to S3

Data S1 to S2

References (39–50)
