# Supplementary material for: De novo design and structure of a peptide-centric TCR mimic binding module
Source: Science. Author manuscript; Available in PMC 2025 Jul 31. (PMC12313176; doi:10.1126/science.adv3813)
Supplement: Supplement [file NIHMS2091935-supplement-Supplement.pdf]

Science
AAAS

# Supplementary Materials for

## *De novo* design and structure of a peptide-centric TCR mimic binding module

Karsten D. Householder, Xinyu Xiang, Kevin M. Jude, Arthur Deng, Matthias Obenaus, Yang Zhao, Steven C. Wilson, Xiaojing Chen, Nan Wang, K. Christopher Garcia*
Correspondence to: kcgarcia@stanford.edu

**The PDF file includes:**

Materials and Methods
Figs. S1 to S6
Tables S1 to S3
Captions for Data S1 to S2

**Other Supplementary Materials for this manuscript include the following:**

Data S1 to S2 [dataS1_all_designs, dataS2_all_peptides]

## Materials and Methods

Cell culture

T2 (ATCC CRL-1992) and Jurkat NFAT-eGFP reporter cells were grown in RPMI 1640 Medium with 1x GlutaMax (Gibco) supplemented with 10% v/v fetal bovine serum (FBS), penicillin-streptomycin (Gibco), MEM-NEAA (Gibco), HEPES (Gibco), Na-Pyruvate (Gibco) and maintained at 37°C with 5% $CO_2$. Expi293F cells were grown in serum-free Expi293 expression media (Thermo) and maintained at 37°C with 5% $CO_2$. BL21 (DE3) *E. coli* (Novagen) were grown in LB media with carbenicillin or kanamycin at 37°C. EBY100 *S. cerevisiae* (ATCC MYA4941) were grown in SDCAA media pH 4.5 at 30°C. The A375 (ATCC CRL-1619) human melanoma cell line was grown in DMEM media with 10% v/v FBS, penicillin-streptomycin (Gibco), 1x GlutaMax (Gibco), Na-Pyruvate (Gibco), $NaHCO_3$ (Gibco) and maintained as adherent cells at 37°C with 5% $CO_2$. After activation, human primary T cells were cultured in RPMI 1640 Medium with 1x GlutaMax (Gibco) supplemented with 100U/mL IL-2 (PeproTech), 10% v/v fetal bovine serum (FBS), penicillin-streptomycin (Gibco), MEM-NEAA (Gibco), HEPES (Gibco), Na-Pyruvate (Gibco) and maintained at 37°C with 5% $CO_2$.

Mini-TCR mimic design

RFdiffusion model weights and code were downloaded from the RFdiffusion GitHub repository (https://github.com/RosettaCommons/RFdiffusion). Backbone structures of four-helix α-helical bundles (80-120 residues) were sampled, and a single optimal scaffold was selected by visual inspection for use as an input backbone in RFdiffusion fold conditioning. The target peptide-MHC structure for NY-ESO-1 HLA-A*02 was created with AlphaFold2. Hotspot residues on the peptide were selected to be Met4, Trp5, Thr7, and Gln8. ProteinMPNN and AlphaFold2 model weights and code were downloaded according to the dl_binder_design Github repository (https://github.com/nrbennet/dl_binder_design). Scaffolds generated by RFdiffusion were then fed into the dl_binder_design pipeline for sequence design and scoring by ProteinMPNN and AlphaFold2. All output designs were ranked from lowest to highest iPAE. Designs were considered hits if their iPAE was less than 10.0.

Bispecific T cell engager design

T cell engagers were designed as C-terminal fusions with mouse serum albumin (MSA) in the pD649 mammalian expression vector, containing an N-terminal hemagglutinin (HA) signal peptide and C-terminal 8x-His tag. The anti-human CD3ε scFv L2K-07 from blinatumomab (*37*) was fused C-terminally to MSA with a seven amino acid Gly-Ser linker, followed by a five amino acid Gly-Ser linker and the mini-TCR mimic.

Yeast display screening by flow cytometry

Gene blocks for the top mini-TCR mimic designs were synthesized with overhangs for C-terminal display on digested pCT3CBN vector. Individual gene blocks and vector were mixed and electroporated as single clones into electrocompetent EBY100 yeast. Electroporation, rescue, expansion, and induction were performed as previously described (*39*). Data was collected on an Accuri C6 flow cytometer (Beckman Coulter). Mini-TCR mimics were stained with AlexaFluor-647-conjugated streptavidin (SA) as the control, 100 nM on-target tetramer (NY-ESO-1 HLA-A*02; Acro Biosystems), or 100 nM off-target tetramer (MART-1 HLA-A*02; Acro Biosystems). Designs that stained for NY-ESO-1 but not MART-1 were considered hits and advanced for further characterization (gating strategy in fig. S4A).

Surface plasmon resonance

A BIAcore Control T100 (GE Healthcare) was used to measure $K_d$ by the multi-cycle kinetics method. Biotinylated pMHC (Acro Biosystems) was immobilized on a SA sensor chip (Cytiva) at 100-140 response units. Purified mini-TCR mimic was injected at varying concentrations (0-156 nM) with 10 mM HEPES, 150 mM NaCl, 0.05% v/v Surfactant P20, pH 7.4 (HBS-P+) (Cytiva) for 60 s at a flow rate of 30 μL/min, then dissociation was measured for 60 s with buffer flow. The signals of reference cells were subtracted from measurements. Data analysis was performed with BIAcore T100 evaluation software.

Protein production and purification

For mini-TCR mimic production, gene blocks were cloned into pETDuet1 vectors with a C-terminal 6x-His tag. Plasmids were then transformed into competent BL21(DE3) *E. coli* and allowed to grow overnight shaking at 37°C in LB starter cultures. The next day, starter culture was added to 1 L of LB media and continued to incubate until OD = 0.6-0.8. Culture was then induced with 0.2 mM isopropyl β-D-1-thiogalactopyranoside (IPTG) (Sigma Aldrich) overnight shaking at 18°C. Cells were then pelleted and lysed with B-PER Bacterial Protein Extraction Reagent lysis buffer (Thermo) according to the manufacturer's protocol and protein was purified with Pierce Nickel-NTA resin (Thermo) followed by Size Exclusion Chromatography (SEC) on a Superdex 200 column (Cytiva) in 20 mM HEPES pH 7.4, 150 mM NaCl (HBS).

For 3M4E5 scFv production, gene blocks were designed to connect the 3M4E5 Fab's variable region heavy and light chains with an 18 amino acid flexible linker, and a C-terminal 8x-His tag. Gene blocks were cloned into pD649 plasmids and then transiently transfected into the Expi 293F mammalian cell line using Expifectamine transfection reagent. Cell supernatant was harvested and purified 96 to 120 hours later with Nickel-NTA resin followed by SEC on a Superdex 200 column in HBS.

For T cell engager production, designed plasmids were transiently transfected into the Expi293F mammalian cell line using Expifectamine transfection reagent. Cell supernatant was harvested and purified 96 to 120 hours later with Nickel-NTA resin followed by SEC on a Superdex 200 column in HBS.

For anti-β2M nanobody production, a gene block of nanobody AD01 (*40*) was cloned into pD649 with an N-terminal HA signal peptide and C-terminal 8x-His tag. The plasmid was transiently transfected into Expi293F cells, and after five days, the supernatant was collected and the protein was batch purified on Ni-NTA resin, followed by SEC on a Superdex 75 column (Cytiva) in HBS. AD01 was stored in flash-frozen aliquots before use.

For production of HLA-A*02 and β2M inclusion bodies, gene blocks were cloned into pETDuet1 vectors with no tags. Plasmids were transformed into competent BL21 BL21(DE3) *E. coli* and allowed to grow overnight with shaking at 37°C in LB starter cultures. The next day, starter culture was added to 1 L of LB media and continued to incubate until OD = 0.5-0.7. Culture was then induced with 1 mM IPTG shaking at 30°C for 3 hours. Cells were pelleted and resuspended in resuspension buffer (50 mM Tris-HCl pH 8.0, 1 mM EDTA, 10 mM DTT). Cell pellets were then lysed with lysis buffer (50 mM Tris-HCl pH 8.0, 1% Triton X-100, 100 mM

NaCl, 10 mM DTT) rotating for 20 minutes at room temperature. Lysate was sonicated and pelleted. Inclusion body pellets were then washed and pelleted three times with detergent-based wash buffer (50 mM Tris-HCl pH 8.0, 0.5% Triton X-100, 1 mM DTT, 100 mM NaCl, 1 mM EDTA). Finally, the preparation was washed with detergent-free wash buffer (50 mM Tris-HCl 8.0, 1 mM EDTA, 1 mM DTT, 0.2 mM PMSF) and pelleted. Inclusion bodies were solubilized with urea buffer (8M urea, 20 mM Tris-HCl pH 8.0, 0.5 mM EDTA, 1 mM DTT) and frozen at -80°C until use.

<u>CAR-T cell transduction</u>

Gene blocks were cloned into MSGV1-CAR retroviral transfer plasmids, which encode the mini-TCR mimic, Myc tag, CD8 transmembrane domain, 4-1BB costimulatory domain, and CD3ζ domain. Virus was produced in the Platinum-GP Retroviral Packaging Cell Line (Cell Biolabs) by transfecting transfer plasmid with RD114 envelope plasmid in 3:1 w/w ratio. The viral supernatant was harvested after 48 and 72 hours. Before transduction, human T cells were activated from peripheral blood mononuclear cells (PBMCs) from the Stanford Blood Center for 24 hours with plate-bound anti-human CD3ε (1 ug/mL, clone OKT-3, BioXCell) and soluble anti-human CD28 (5ug/mL, clone 9.3, BioXCell), and 100 U/mL IL-2 (Peprotech). Activated T cells were transduced on plates coated with Retronectin (25 ug/mL, Takara) and loaded with virus by spinfection. Cells were then further expanded for 5 more days with 100 U/mL IL-2 before use in co-culture assays.

<u>Refolding and purifying peptide-MHC</u>

Peptides for refolding were synthesized by Elim Biopharm. Refolding buffer was prepared stirring at 4°C (100 mM Tris-HCl pH 8.0, 2 mM Na EDTA, 400 mM L-Arginine-HCl, 0.5 mM oxidized glutathione, 5 mM reduced glutathione) with 10 mg of NY-ESO-$1_{157\text{-}165\ (C9V)}$ peptide. 10 mg of each solubilized inclusion body was combined and slowly added to refolding buffer (*41*). The refolding mixture was then dialyzed four times against 10 mM Tris-HCl buffer. Refolded pMHC was purified by SEC Superdex 200, followed by MonoQ (GE Healthcare) columns.

<u>X-ray crystallography of complex</u>

Mini-TCR mimic, refolded NY-ESO-1 pMHC, and AD01 nanobody were complexed in a 3:1:1 molar ratio and incubated at 4°C overnight with 1:1000 (w/w) carboxypeptidases A and B. The complex was purified by a SEC Superdex 200 column and the co-eluting fractions were confirmed by SDS-PAGE gel. Purified complex was concentrated to 10.8 mg/mL and crystallized using the Index screen (Hampton Research) in 0.2 M ammonium acetate, 100 mM bis-tris pH 5.5, and 25% PEG 3350. Crystals were cryoprotected by addition of 30% glycerol and flash cooled in liquid nitrogen. Diffraction data were collected at Advanced Light Source (ALS) beamline 8.2.1. Initial data processing with XDS and pointless (*42*, *43*) suggested that the space group was $P2_12_12$ with cell dimensions a = 95.4 Å, b = 98.5 Å, c = 77 Å. Structure solution by molecular replacement using Phaser (*44*) with AlphaFold2 models (*34*) of the mini-TCR mimic and the extracted crystal structure of NY-ESO-1 pMHC (PDB: 3HAE). One copy of the complex was found in the asymmetric unit, but the electron density maps were barely interpretable. Reinspection of the diffraction data revealed a true space group of $P2_12_12_1$ with a doubled c axis of 155.7 Å. A translational non-crystallographic symmetry vector parallel to the c-axis was found to have caused very weak reflections for l = 2n. Repeating the molecular

replacement procedure identified two complexes in the asymmetric unit and produced interpretable electron density maps (fig. S5). The model was built and refined in iterative cycles of interactive and automated refinement using Coot (*45*) and Phenix (*46*) using torsional NCS restraints (*47*). TLS groups were assigned using TLSmd (*48*). The final structure had 98.71% of residues in the favored region of the Ramachandran plot, with no outliers. Crystallographic data and refinement statistics are reported in Table S1. Crystallographic software for this project was installed and configured using SBGrid (*49*). PDBePISA software was used to calculate buried surface area for the mini-TCR mimic, 3M4E5 Fab, and 1G4 TCR and to confirm, count, and compare key interactions observed in the crystal structures (table S2) (*50*).

T2 peptide pulsing assays

Peptides for pulsing assays were ordered from Elim Biopharm. 0.5E6 T2 cells per well were plated into a 96-well plate and 100 µM peptide was prepared in serum-free RPMI 1640 with 1x Glutamax (Gibco). Peptides were added to T2 and allowed to incubate at 37°C with 5% $CO_2$ for 2 hours. Excess peptide was washed two times with serum-free media. Pulsed cells were then stained with 1 µM mini-TCR mimic or 3M4E5 scFv in PBS for 20 minutes on ice, followed by washing and staining with FITC-conjugated anti-His tag antibody (50:1, Biolegend) for 20 minutes on ice. Plates were washed and the geometric mean fluorescence intensity (MFI) was measured using a CytoFlex flow cytometer (Beckman Coulter) (gating strategy in fig. S4B).

T cell engager signaling assays with Jurkats

T2 cells were pulsed with 10 µM peptide for 2 hours as previously described. 0.04E6 T2 and 0.04E6 Jurkat NFAT-eGFP per well were added to a 96-well plate. Starting from 100 nM mini-TCRm engager, seven 5:1 serial dilutions were added to each well, including controls with no engager. Co-cultures were incubated for 16-18 hours at 37°C with 5% $CO_2$. The next day, cells were washed and stained for 20 minutes on ice with Zombie Violet Live/Dead (500:1, Biolegend), PE-conjugated CD3 antibody (50:1, UCHT1 clone, Biolegend), and APC/Cy7-conjugated anti-CD69 antibody (50:1, Biolegend). Data were collected on a CytoFlex flow cytometer with compensation applied. Jurkat NFAT-eGFP cells were identified as the $SSC_{lo}$ Live+ CD3+ population. Compared to controls with either no peptide pulsing or no engager, the percentage of these cells that were CD69+ NFAT-eGFP+ were considered activated Jurkat cells (fig. S4C).

T cell engager cytotoxicity assays with primary T cells

Human PBMCs were obtained from the Stanford Blood Center and plate-activated for 48 hours with Ultra-LEAF anti-human CD3ε antibody (1ug/mL, clone OKT-3, Biolegend), soluble Ultra-LEAF anti-human CD28 antibody (5ug/mL, clone 9.3, Biolegend), and 50U/mL IL-2 (Peprotech). Remaining T cells were expanded for an additional 5 days with 100 U/mL IL-2 before resting for 18 hours in media overnight. On the day of the assay, T2 cells were pulsed with 10 µM peptide for 2 hours as previously described. 0.04E6 T2 and 0.04E6 rested T cells per well were added to a 96-well plate. Starting from 300 nM mini-TCRm engager, seven 3:1 serial dilutions were added to each well, including negative controls with no peptide or no engager and an anti-CD3 positive control. The co-culture was incubated overnight for 16 hours, after which point, 500:1 Protein Transport Inhibitor Cocktail (eBioscience) was added for 4 more hours. At 20 hours of co-culture, cells were collected and washed, followed by Zombie Violet (Thermo) live/dead staining for 10 minutes and surface antibody staining for 30 minutes on ice with 50:1

anti-CD8 PE (Biolegend) and anti-CD69 Alexa Fluor 488 (Biolegend). After washing, cells were fixed and permeabilized according to the BD Cytofix/Cytoperm Fixation/Permeabilization Kit instructions and reagents. 50:1 anti-IFNγ PE-Dazzle-594 (Biolegend), anti-Granzyme B Alexa Fluor 700 (Biolegend), and anti-IL-2 PE/Cyanine7 antibodies (Biolegend) were used for intracellular staining for 1 hour. Cells were washed with BD Perm/Wash buffer and data were collected on a CytoFlex flow cytometer with compensation applied (gating strategy in fig. S4D).

CAR-T cell cytotoxicity assays

CAR-T cells were transduced and expanded as previously described. Mock T cells that were untransduced but activated were included as controls. 0.05E6 A375 were dispensed in each well of a flat bottom 96-well plate about 4 hours before starting the co-culture, to allow them to adhere. 0.05E6 CAR-T cells were then added to each well and incubated for 48 hours at 37°C with 5% $CO_2$. 500:1 Protein Transport Inhibitor Cocktail (eBioscience) was added for another 4 hours. Cells were collected and washed, followed by Zombie Violet (Thermo) live/dead staining for 10 minutes and surface staining for 30 minutes on ice (100 nM tetramer with lab-made streptavidin-647 and NY-ESO-1 HLA-A*02, Acro Biosystems; 50:1 anti-CD69 Alexa Fluor 488, Biolegend). After washing, cells were then fixed and permeabilized according to the BD Cytofix/Cytoperm Fixation/Permeabilization Kit instructions and reagents. 50:1 anti-IFNγ Alexa Fluor 700 (Biolegend) and anti-Granzyme B PE-Cyanine7 antibodies (Biolegend) were used for intracellular staining for 1 hour. Cells were washed with BD Perm/Wash buffer and data were collected on a CytoFlex flow cytometer with compensation applied (gating strategy in fig. S4E).

Selecting off-target peptides by Hamming distance

We downloaded all 9mer HLA-A*02 peptides from the MHC Motif Atlas and used a short Python script that calculates the Hamming distance for each peptide (compared to the natural NY-ESO-1 peptide – SLLMWITQC) to output a CSV file ranking this dataset by lowest to highest Hamming distance. This yielded six peptides with the lowest Hamming distance of four. Five of the six peptides were found in the human proteome and therefore included in the dataset. Next, we calculated the Hamming distance for peptides with the Met-Trp motif at positions 4 and 5 and sorted the dataset. This returned only two exact matches (peptides with a Hamming distance of 0). Both were found in the human proteome and were therefore added to our final dataset.

Scoring off-target peptides with ProteinMPNN

Using our crystal structure, we generated a positional matrix of ProteinMPNN negative log-likelihood values for all twenty amino acids at each position in the peptide backbone. For each off-target peptide's amino acid sequence, we calculated the sum of negative log-likelihood values at each residue position. We performed ten replicates for each off-target peptide and averaged these runs. Then, we repeated this procedure for the original NY-ESO-1 peptide sequence and subtracted this value from each off-target peptide's score. This final value is what we term the "off-target score". Off-target scores less than 0 indicate that the new peptide fits the crystal structure backbone better than NY-ESO-1, whereas a score greater than 0 indicates a worse fit. Peptides were ranked by lowest to highest off-target scores.

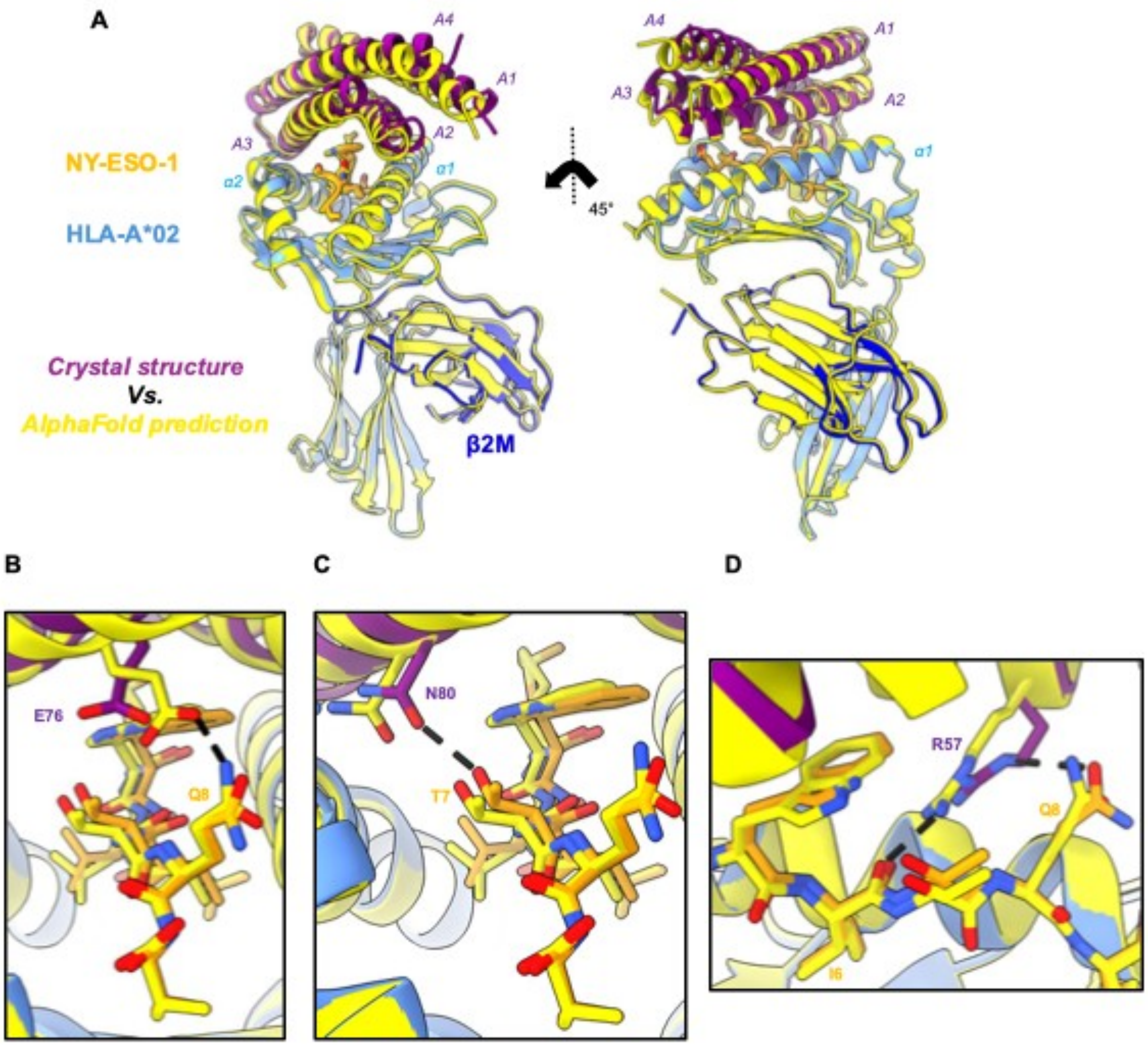


**Fig. S1. Comparison of crystallographic peptide contacts to the AlphaFold prediction.**
**(A)** Front and side view of the crystal structure aligned with the AlphaFold prediction (yellow). **(B)** Hydrogen bond in AlphaFold prediction between Glu76 of mini-TCRm and Gln8 of NY-ESO-1 (yellow). No interaction in crystal structure (purple and gold). **(C)** Hydrogen bond in crystal structure between Asn80 of mini-TCRm (purple) and Thr7 of NY-ESO-1 (gold). No interaction in AlphaFold prediction (yellow). **(D)** Hydrogen bond in crystal structure between Arg57 on mini-TCRm (purple) and Gln8 on NY-ESO-1 (gold). AlphaFold prediction makes a hydrogen bond between Arg57 and Ile6's backbone (yellow).

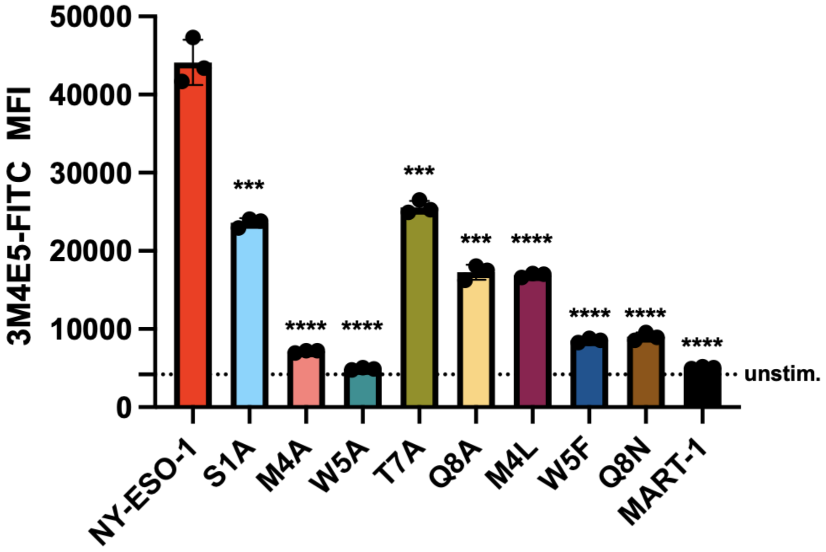


**Fig. S2. Mutation scanning the 3M4E5 anti-NY-ESO-1 scFv.**
Flow cytometry assay of the 3M4E5 scFv (PDB ID 3HAE) staining T2 cells pulsed with mutation scanned peptides (see fig. S4B for same gating strategy as mini-TCR mimic staining; see data S2 for peptide sequences). MART-1 and no peptide were used as negative controls. Data represent geometric MFI from technical replicates (bars are mean ± SD, $n$=3, $N$=2). Statistical significance calculated relative to NY-ESO-1 (***$P < 0.001$, ****$P < 0.0001$, unpaired Student’s t-tests).

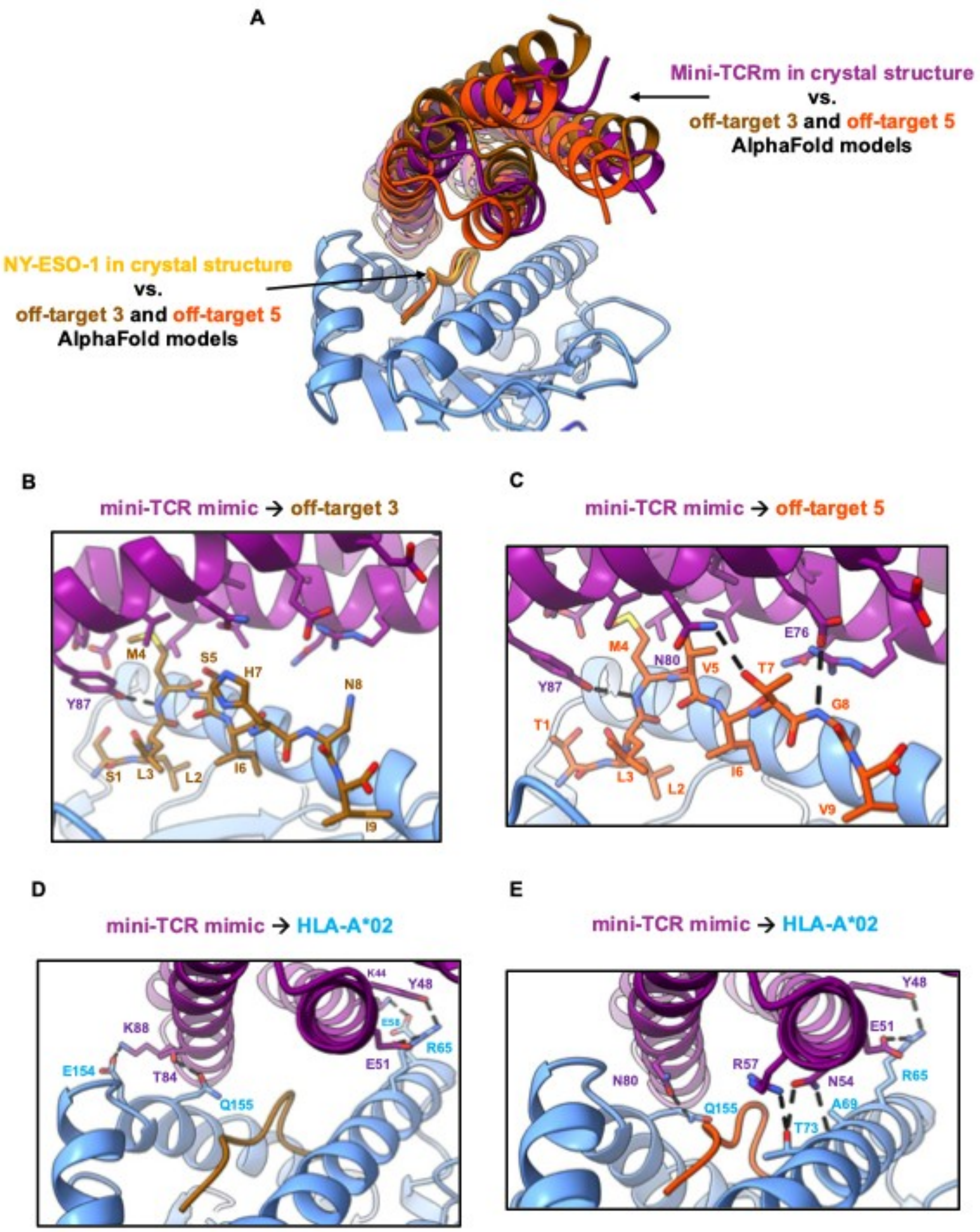


**Fig. S3. Comparison of the crystal structure to off-target AlphaFold predictions.**

**(A)** Alignment of mini-TCRm in the crystal structure (purple) docked over the NY-ESO-1 peptide (gold), versus the predicted docking geometry of the mini-TCRm over off-target 3 peptide (brown) and off-target 5 (orange). **(B)** Hydrogen bond in AlphaFold prediction between Tyr87 of mini-TCRm (purple) and the Met4 backbone of the off-target 3 peptide (brown). **(C)** Hydrogen bonds in AlphaFold prediction between Tyr87, Asn80, and Glu76 of mini-TCRm (purple) and Met4 backbone, Thr7, and Gly8 backbone of the off-target 5 peptide (orange). **(D)** Hydrogen bonds to HLA-A*02 in the AlphaFold prediction for off-target 3 (brown). **(E)** Hydrogen bonds to HLA-A*02 in the AlphaFold prediction for off-target 5 (orange).

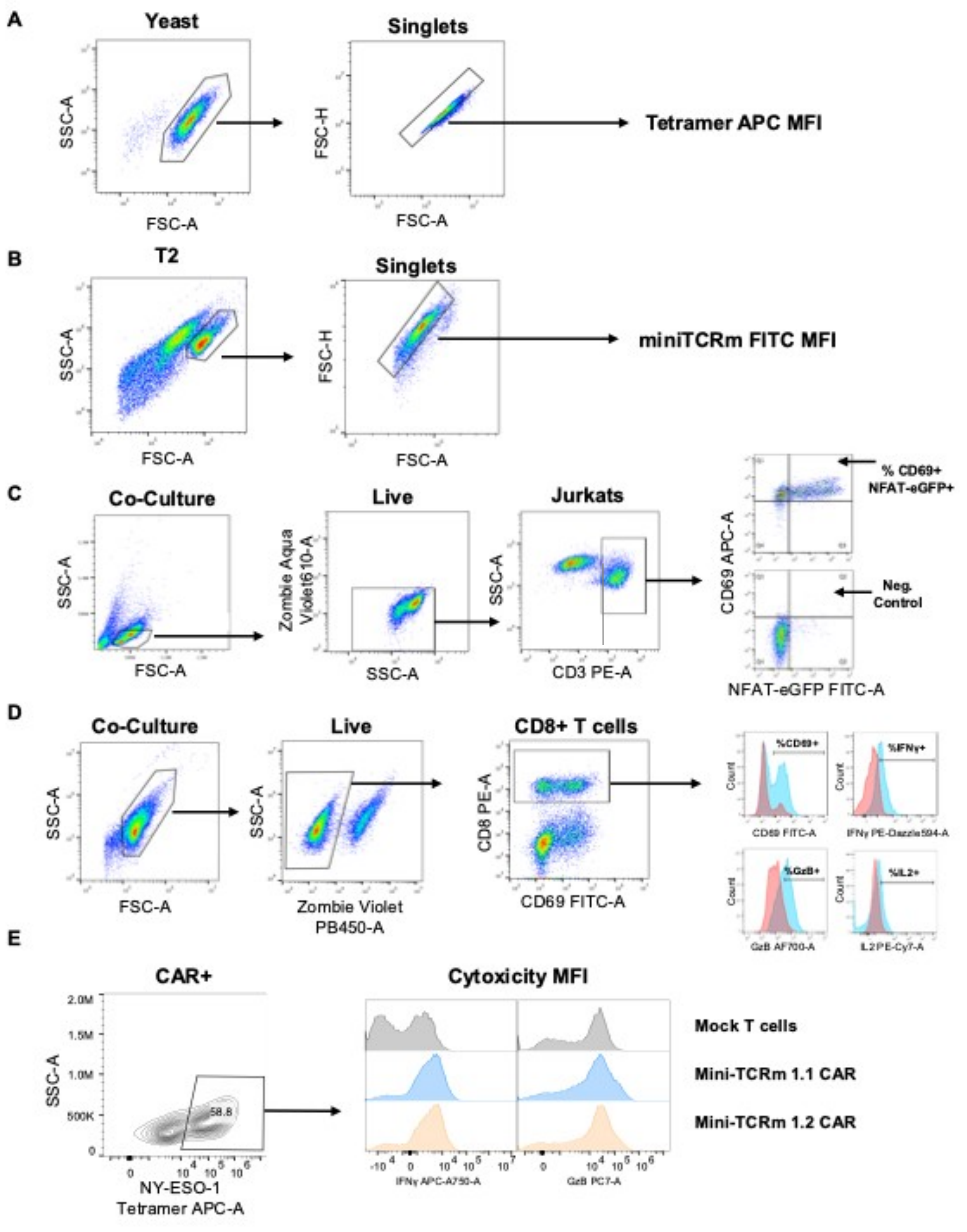


**Fig. S4. Gating strategies and examples for flow cytometry assays.**

**(A)** Gating strategy for yeast display of mini-TCR mimic clones. Singlets were evaluated by peptide-MHC tetramer geometric mean fluorescence intensity (MFI). **(B)** Gating strategy for T2 assays (both mutation scanning and off-target assays). Singlets were evaluated by mini-TCR mimic geometric mean fluorescence intensity (MFI). **(C)** Gating strategy for T2-Jurkat co-culture assay with T cell engagers. CD3+ Jurkats were further analyzed by CD69 and NFAT-eGFP values. A four-quadrant panel gated on the negative control identified the percentage of Jurkat cells that were CD69+ NFAT-eGFP+ double positive. **(D)** Gating strategy for T2 and

primary human T cells. The percentage of cells positive (blue) for each marker (CD69, IFNγ, Granzyme B (GzB), IL2) were identified by gating relative to the negative control (red), within the CD8+ T cell population. **(E)** Gating strategy for mini-TCR mimic CAR-T assay. CAR+ cells were NY-ESO-1 HLA-A*02 tetramer positive, and this population was used to plot CD69, IFNγ, and GzB geometric mean fluorescence intensity (MFI).

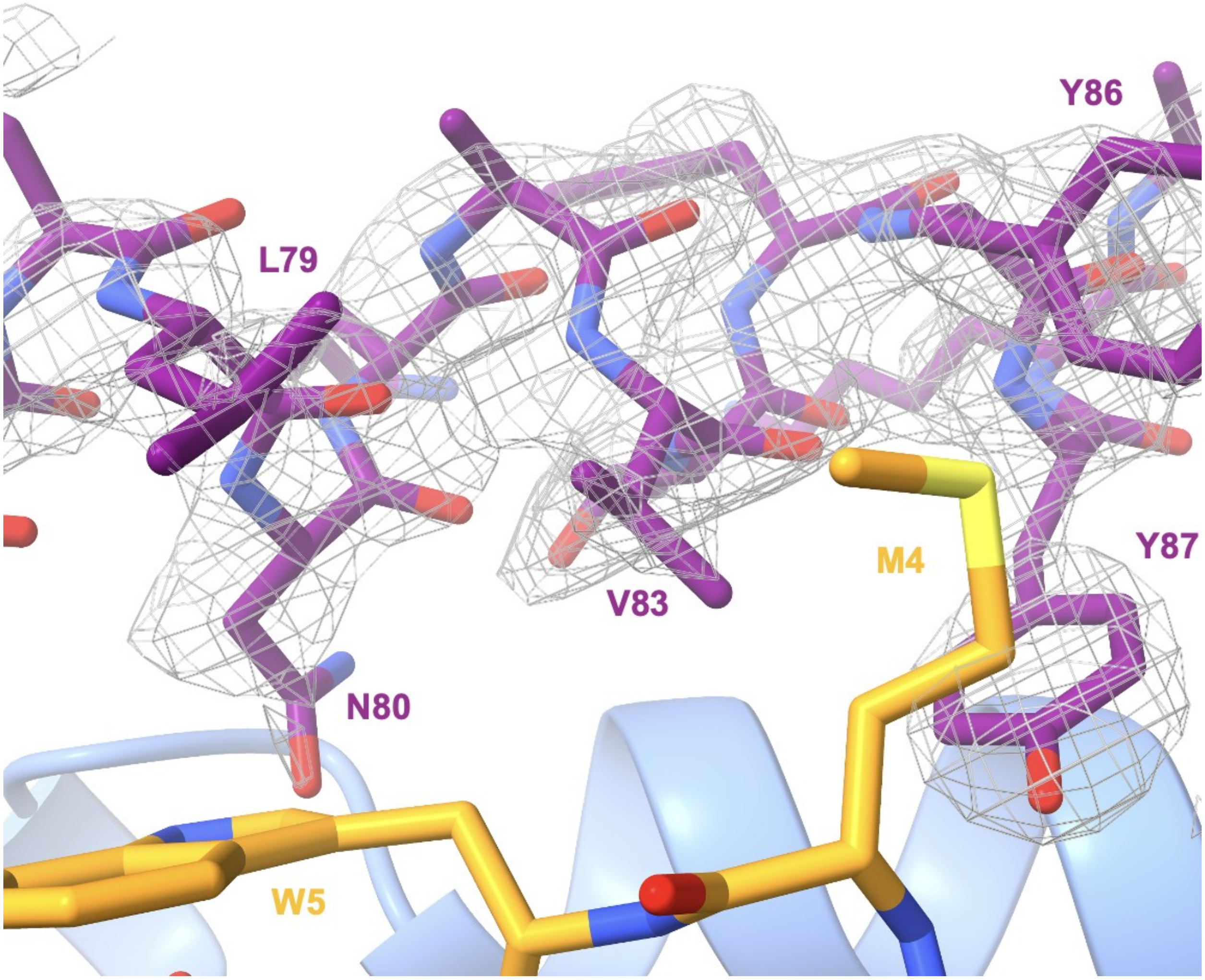


**Fig. S5. Fitting of the mini-TCRm/pMHC complex structure into the electron density map.** 2mFo-DFc electron density map contoured at 1σ (gray) around mini-TCRm A3 helix (purple). Key interacting mini-TCRm residues are labeled. NY-ESO-1 positions Met4 and Trp5 (gold) and the α2 helix of HLA-A*02:01 (light blue) are shown as sticks and cartoon, respectively.

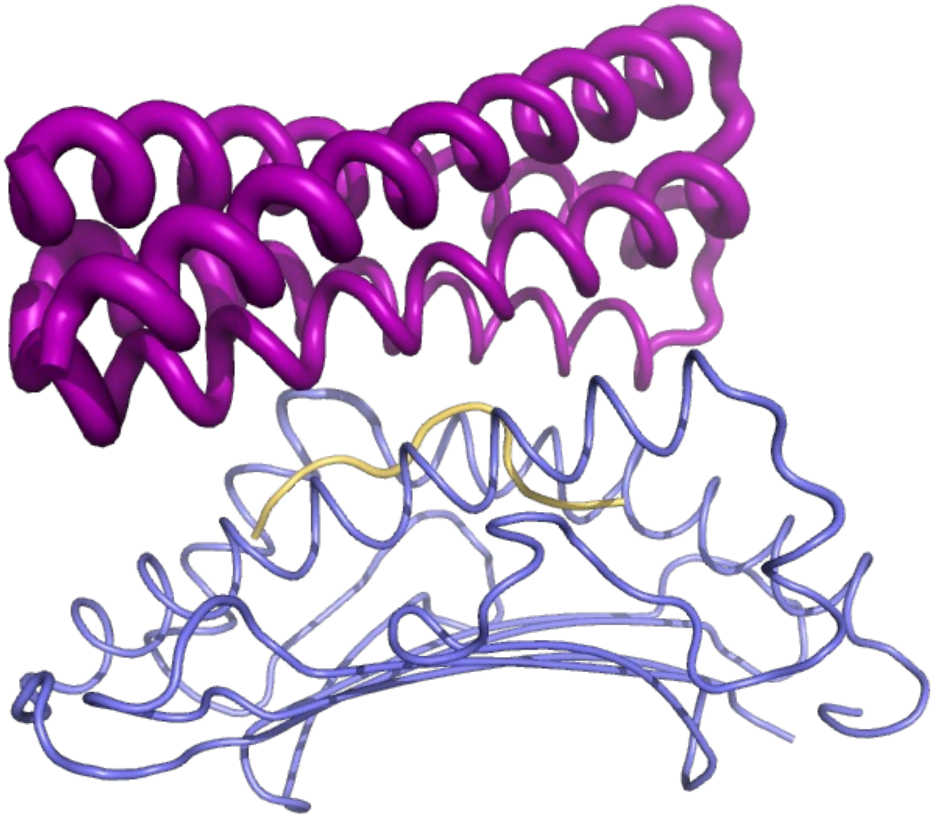

**Fig. S6. Putty representation of B-factors.**
Side view of the mini-TCR mimic (purple) bound to the NY-ESO-1 peptide (yellow) and HLA-A*02 complex (lavender). The thickness of the cartoon represents the relative B-factor at each residue position. Regions with elevated B-factors are distal to the peptide-MHC interface.

**Table S1. Crystallographic Data and Refinement Statistics.**

| | Mini-TCR mimic + NY-ESO-1 + A*02:01 + AD-01 Nb |
|---|---|
| **Wavelength (Å)** | 1.00003 |
| **Resolution range (Å)** | 49.27 - 2.05 (2.12 - 2.05) |
| **Space group** | P $2_12_12_1$ |
| **Unit cell (Å, °)** | 95.45 98.53 155.74 90 90 90 |
| **Total reflections** | 2159405 (218759) |
| **Unique reflections** | 92563 (9127) |
| **Multiplicity** | 23.3 (24.0) |
| **Completeness (%)** | 99.1 (100.0) |
| **Mean I/sigma(I)** | 8.4 (1.4) |
| **Wilson B-factor ($Å^2$)** | 33.17 |
| **R-merge** | 0.325 (3.471) |
| **R-meas** | 0.332 (3.545) |
| **R-pim** | 0.068 (0.716) |
| **CC1/2** | 0.999 (0.349) |
| **Reflections used in refinement** | 91895 (9127) |
| **Reflections used for R-free** | 1384 (133) |
| **R-work** | 0.254 (0.398) |
| **R-free** | 0.267 (0.413) |
| **Number of non-hydrogen atoms** | 10482 |
| **macromolecules** | 10116 |
| **other heteroatoms*** | 96 |
| **solvent** | 270 |
| **Protein residues** | 1258 |
| **RMS(bonds) (Å)** | 0.003 |
| **RMS(angles) (°)** | 0.53 |
| **Ramachandran favored (%)** | 98.55 |
| **Ramachandran outliers (%)** | 0.00 |
| **Rotamer outliers (%)** | 2.43 |

| | |
|---|---|
| **Clashscore** | 4.95 |
| **Average B-factor (Å$^2$)** | 60 |
| **HLA-A*02 (chain A)** | 52 |
| **B-2-microglobulin (chain B)** | 38 |
| **NY-ESO-1 peptide (chain C)** | 56 |
| **Mini-TCRm (chain D)**** | 126 |
| **AD-01 Nanobody (chain E)** | 44 |
| **HLA-A*02 (chain F)** | 54 |
| **B-2-microglobulin (chain G)** | 38 |
| **NY-ESO-1 peptide (chain H)** | 53 |
| **Mini-TCRm (chain I)**** | 112 |
| **AD-01 Nanobody (chain J)** | 44 |
| **macromolecules** | 61 |
| **other heteroatoms*** | 54 |
| **solvent** | 42 |

Statistics for the highest-resolution shell are shown in parentheses.
*Other heteroatoms are from glycerol, which was used as cryoprotectant during data collection and is visible in the electron density.
**The largest B-factor values on the mini-TCR mimic are distant from the interface (see fig. S6).

**Table S2. PDB-PISA Interface Analysis of Crystal and AlphaFold3 Structures.**

| Structure | Buried Surface Area | # h-bonds | # salt bridges |
|---|---|---|---|
| *Crystal mini-TCRm* | 1216 Å (384, 832) | 8 (3, 5) | 1 (0, 1) |
| *AlphaFold mini-TCRm* | 1311 Å (388, 922) | 12 (3, 9) | 5 (0, 5) |
| *3M4E5 Fab* | 1055 Å (405, 649) | 10 (6, 4) | 1 (0, 1) |
| *1G4 TCR* | 1207 Å (441, 766) | 12 (5, 7) | 0 (0, 0) |
| *AlphaFold Off-target 3* | 940 Å (270, 669) | 6 (1, 5) | 3 (0, 3) |
| *AlphaFold Off-target 5* | 1213 Å (308, 904) | 10 (3, 7) | 1 (0, 1) |

The first and second items in the parentheses are peptide and HLA-A*02 -specific contacts with the binder, respectively.

**Table S3. Residue-residue Contacts in Crystal Structure versus AlphaFold3 Prediction**. Model of miniTCRm/A*02:01/NY-ESO complex using 3.8 Angstrom distance threshold applied with methyl group van der Waals radius.

| miniTCRm residue | A*02:01 residue | Crystal Structure | Design | Contact type |
|---|---|---|---|---|
| Leu 47 | Lys 66 | + | + | sc-sc |
| Tyr 48 | Arg 65 | + | - | mc-sc |
| Tyr 48 | Arg 65 | + | + | sc-sc |
| Val 50 | Ala 69 | + | + | sc-sc |
| Val 50 | Arg 65 | + | - | sc-sc |
| Glu 51 | Arg 65 | + | + | sc-sc |
| Asn 54 | Ala 69 | + | + | sc-sc |
| Asn 54 | Ala 69 | - | + | sc-mc |
| Asn 54 | Thr 73 | + | + | sc-sc |
| Arg 57 | Arg 65 | + | - | sc-sc |
| Arg 57 | Val 76 | + | - | sc-sc |
| Arg 57 | Thr 73 | + | + | sc-sc |
| Val 58 | Arg 75 | + | - | sc-sc |
| Val 58 | Gln 72 | - | + | sc-sc |
| Asn 80 | Gln 155 | + | - | sc-sc |
| Asn 80 | Ala 150 | + | + | sc-mc |
| Asn 80 | Gln 155 | + | + | sc-sc |
| Asn 80 | Ala 150 | + | + | sc-mc |
| Asn 80 | His 151 | + | + | sc-mc |
| Asn 80 | Gln 155 | + | - | mc-sc |
| Lys 81 | Glu 154 | - | + | sc-sc |
| Val 83 | Gln 155 | + | + | sc-sc |
| Thr 84 | Gln 155 | + | + | sc-mc |
| Thr 84 | Gln 155 | + | + | sc-sc |
| Tyr 87 | Ala 158 | + | + | sc-sc |
| Tyr 87 | Thr 163 | + | + | sc-sc |
| Tyr 87 | Tyr 159 | + | + | sc-sc |
| Tyr 87 | Gln 155 | - | + | sc-sc |
| Lys 88 | Glu 154 | - | + | sc-sc |
| Lys 90 | Thr 163 | + | - | sc-sc |
| Lys 90 | Glu 166 | + | - | mc-sc |
| Leu 91 | Gly 162 | + | - | sc-mc |
| Leu 91 | Glu 166 | - | + | sc-sc |

| miniTCRm residue | NY-ESO residue | Crystal Structure | Design | Contact type |
|---|---|---|---|---|
| Leu 46 | Met 4 | + | - | mc-sc |
| Leu 46 | Met 4 | + | + | sc-sc |
| Leu 47 | Met 4 | + | - | sc-sc |
| Leu 47 | Met 4 | + | - | mc-sc |
| Val 50 | Met 4 | + | + | sc-mc |
| Val 50 | Trp 5 | + | - | sc-sc |
| Val 53 | Trp 5 | - | + | sc-sc |
| Asn 54 | Trp 5 | + | + | sc-sc |
| Arg 57 | Trp 5 | + | + | sc-sc |
| Arg 57 | Ile 6 | + | + | sc-mc |
| Arg 57 | Thr 7 | - | + | sc-mc |
| Arg 57 | Gln 8 | + | + | sc-mc |
| Arg 57 | Gln 8 | + | + | sc-sc |
| Glu 76 | Trp 5 | + | + | sc-sc |
| Glu 76 | Thr 7 | + | + | sc-sc |
| Glu 76 | Gln 8 | - | + | sc-sc |
| Leu 79 | Trp 5 | - | + | sc-sc |
| Asn 80 | Thr 7 | + | - | sc-sc |
| Tyr 86 | Met 4 | - | + | sc-sc |
| Tyr 87 | Leu 3 | + | + | sc-mc |
| Tyr 87 | Leu 3 | + | + | sc-sc |
| Tyr 87 | Met 4 | + | + | sc-mc |
| Tyr 87 | Met 4 | + | + | sc-sc |

+ contact observed
- contact not observed
sc: side chain
mc: main chain

**Data S1. dataS1_all_binders.xlsx**
Excel file containing all amino acid sequences and associated metrics for the *de novo* TCR mimics assessed in this work. Includes a comprehensive list of binders from the 600 design scaffold search, and all 500 sequences for each of our top four scaffold hits (2000 designs total).

**Data S2. dataS2_all_peptides.xlsx**
Excel file containing all amino acid sequences and associated metrics for the peptides assessed in this work. Includes a comprehensive list of on-target peptides, mutant peptides, and all off-target peptides.
